# Relationship between lower eyelid epiblepharon and epicanthus in Korean children

**DOI:** 10.1371/journal.pone.0187690

**Published:** 2017-11-21

**Authors:** Dong Hoon Shin, Kyung In Woo, Yoon-Duck Kim

**Affiliations:** Department of Ophthalmology, Samsung Medical Center, Sungkyunkwan University School of Medicine, Seoul, Korea; Public Library of Science, FRANCE

## Abstract

**Purpose:**

This study aimed to determine the relationship between lower eyelid epiblepharon and epicanthus in Korean children.

**Methods:**

We performed a retrospective review of the medical records and preoperative photographs of 119 patients. These patients were aged 18 years or younger who underwent lower lid epiblepharon repair from January 2010 to December 2012. We also included 119 age- and sex-matched controls.

**Results:**

The mean age of the patients was 5.7 years (range: 2–16 years) in both groups. The presence of an epicanthal fold was common in each group (98.3%). The inner intercanthal distance/interpupillary distance (IICD/IPD) ratio was significantly greater in the epiblepharon group than in the control group (mean: 0.70 vs 0.67, p<0.001), especially in those aged 5 years or older. The IICD/outer intercanthal distance ratio was also greater in the epiblepharon group than in the control group (mean: 0.48 vs 0.46, p<0.001). The IICD/IPD ratio decreased with growth in the control group, but it did not decrease with growth in the epiblepharon group. The configuration of the epicanthus, in which the upper skin fold formed a confluent fold with the lower eyelid, had a higher prevalence in the epiblepharon group than in the control group (p = 0.001), especially in those aged 5 years or older.

**Conclusion:**

In early childhood (<5 years), the relationship between epicanthus and epiblepharon cannot be established because of the high prevalence of epicanthus in children with or without epiblepharon. In children aged ≥5 years, persistent epicanthus was related to severe epiblepharon formation requiring corrective surgery.

## Introduction

Epiblepharon is a common eyelid anomaly in Asian children, and often involves lower lids bilaterally [1,2]. In this condition, a horizontal fold of the skin and pretarsal orbicularis override the lid margin, causing the eyelashes to turn inwards. Additionally, keratopathy may develop because of prolonged corneal contact by lashes or frequent rubbing of the eyes.

There are various possible etiologies of epiblepharon. Some authors consider that the skin and pretarsal orbicularis muscle are weakly attached to the tarsus below, thus raising a skin fold near the lid margin and pushing the eyelashes toward the cornea [1,2]. Others have suggested failure of interdigitation of septae in the subcutaneous plane, failure of the lid retractor to gain access to the skin, and hypertrophy of the orbicularis muscle as possible causes [3–10]. However, no definite consensus has been reached on the etiology of epiblepharon. One of these hypotheses, hypertrophy of the orbicularis muscle, was not supported by a histological study [9].

The epicanthus is an eyelid skin fold on the medial aspect of the eye that covers the lacrimal lake [11]. The epicanthus descends over the lacrimal lake to attach to the medial aspect of the lower eyelids. The epicanthal fold is evident in East Asian populations, and in Asia, its incidence ranges between 40% and 90% [12,13]. However, in non-Asian individuals, the epicanthal fold is present in only 2% to 5% of the general population [12,13].

Epiblepharon and epicanthal fold are common eyelid features in Asian children. The lash/corneal touch in lower epiblepharon is generally prominent at the medial side of the eyelid [7,8]. However, none of the above-mentioned hypothetical pathophysiological mechanisms of lower epiblepharon can explain the medial predisposition of epiblepharon. Therefore, this study aimed to investigate the relationship between lower eyelid epiblepharon and epicanthus.

## Materials and methods

The Samsung Medical Center Institutional Review Board approved the retrospective review, and the study complied with the tenets of the Declaration of Helsinki. Written informed consent was provided by the children’s parents or guardians for their clinical records and facial photographs to be used in this study for analysis and for publication.

We retrospectively reviewed 119 Korean children who underwent lower eyelid epiblepharon repair by a single surgeon (K.I.W.) at the Samsung Medical Center between January 2010 and December 2012.

Surgical criteria included the presence of lash-corneal touch due to epiblepharon in the primary position with punctate corneal erosion or severe irritative symptoms. Consecutive patients aged ≤18 years were included. For each patient with epiblepharon, we selected an age and sex-matched control patient from among those who underwent strabismus surgery for intermittent exotropia. To select matched cases, the surgical log of strabismus surgery at Samsung Medical Center was searched from January 2010 to December 2012. Matches were made based on age and sex. When manifest exotropia or esotropia was noted on a photograph, the corresponding patient was replaced with another patient with no visible deviation. The exclusion criteria were as follows: lack of a preoperative photograph, a history of previous trauma or eyelid surgery, Down syndrome, and a congenital craniofacial anomaly. Age and sex and the presence of an upper eyelid crease were noted.

Epicanthus was defined as present when a skin fold covered the medial canthal angle and hid the caruncle vertically. To quantify the severity of epicanthus, the ratio of the inner intercanthal distance (IICD) to interpupillary distance (IPD) (IICD/IPD ratio) and the ratio of the IICD to outer intercanthal distance (OICD) (IICD/OICD ratio) were calculated. The prevalence of epicanthus, severity of epicanthus (as determined by the IICD/IPD and IICD/OICD ratios), configuration of epicanthus, and the prevalence of an upper eyelid crease in the epiblepharon and control groups were compared.

**Fig 1:**
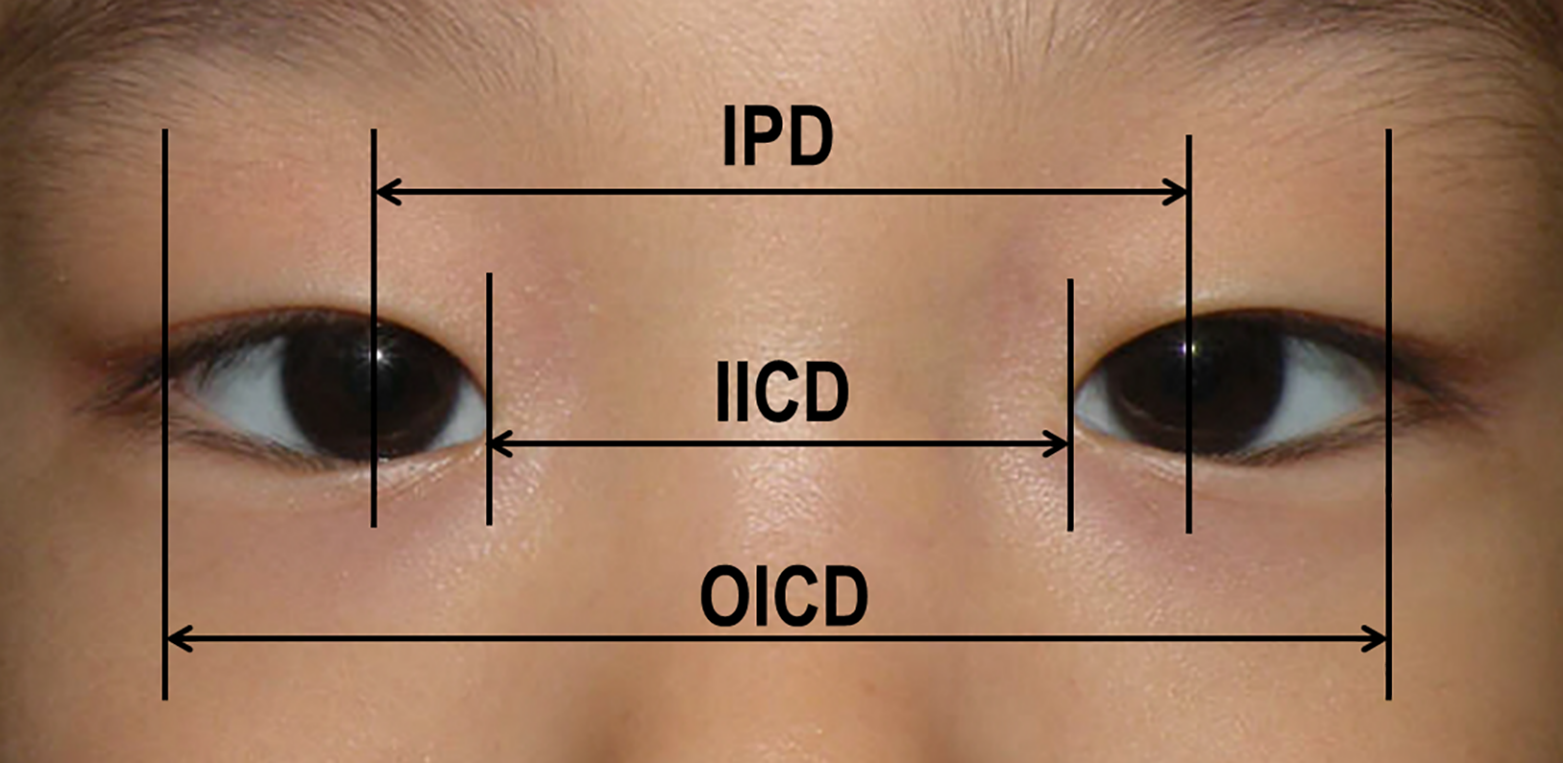
The severity of epicanthus was determined using the inner intercanthal distance (IICD) to interpupillary distance(IPD) ratio and the IICD to outer intercanthal distance (OICD) ratio.

The IICD, OICD, and IPD were measured on digital photographs that were taken before surgery (Fig 1). A single blinded observer measured the IICD, OICD, and IPD using ImageJ (NIH, Bethesda, MD, USA) on magnified images. Measurements were standardized using by assuming a horizontal corneal diameter of 11.5 mm.

**Fig 2:**
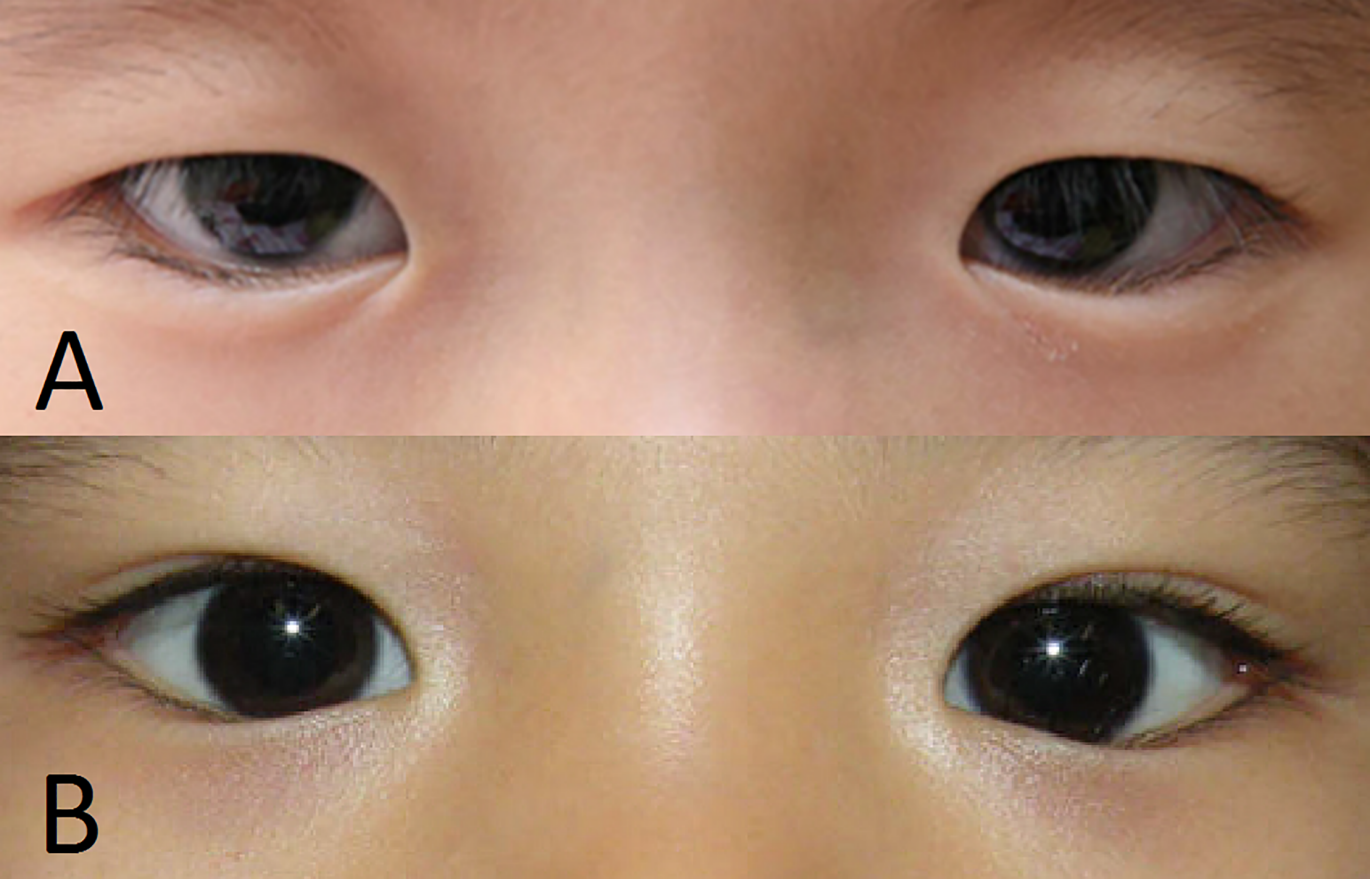
Types of epicanthus. (A) In type 1, a skin fold from the upper eyelid covers the medial canthal angle as it approaches the lower eyelid, creating a crescent-shaped fold onto the lower eyelid. (B) In type 2, a skin fold curves over the medial canthal angle and forms a confluent fold with the lower eyelid at the interpalpebral region.

Epicanthal fold configuration was categorized according to its insertion pattern onto the lower eyelids. In type 1, a skin fold from the upper eyelid was inserted onto the lower eyelid inferior to the lower eyelid margin (Fig 2A). In type 2, the skin fold from the upper eyelid formed a confluent fold with the skin fold from the lower eyelid in the interpalpebral region (Fig 2B). We also assessed correlations between sex or age and epicanthal fold configuration in both groups.

After a pilot study, we calculated that a sample of 44 patients was required to detect a difference in the IICD/IPD ratio between two groups. We had 80% power and a two-sided type I error of 0.05, allowing for a 10% rate of crossover between groups. The IICD/IPD and IICD/OICD ratios of the epiblepharon and control groups were compared using the independent samples t-test. Pearson’s correlation coefficients were used to access correlations between age, the IICD/IPD ratio, and the IICD/OICD ratio in both groups. Univariate analysis was conducted using the chi-square test to compare epicanthal fold configurations between the groups. *P* values of <0.05 were considered statistically significant, and analyses were performed using SPSS for Windows (version 18.0, SPSS Inc., Chicago, IL).

## Results

The epiblepharon group included 58 males and 61 females with a mean age of 5.7±2.8 years (range, 2–16 years). An identical number of age- and sex-matched controls were also enrolled. Epicanthal folds were common in each group (117/119, 98.3%). Only two children in each group had no epicanthal fold.

**Fig 3:**
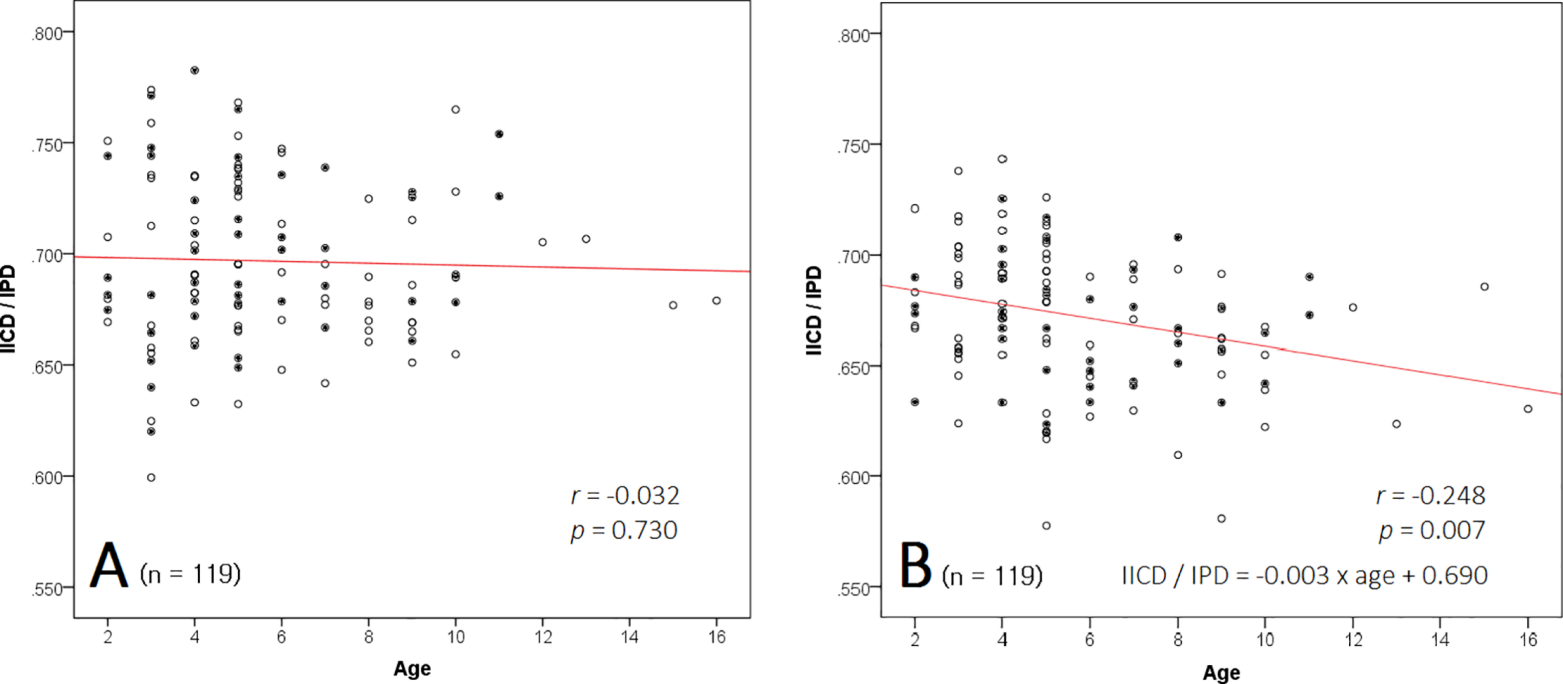
**IICD/IPD ratios were not correlated with age in the epiblepharon group (A), but were negatively correlated with age in the control group (B).** IICD: inner intercanthal distance; IPD: interpupillary distance. Filled circles: males; empty circles: females.

The mean IICD/IPD ratio was greater in the epiblepharon group than in the control group (0.70±0.04 vs. 0.67±0.04, p<0.001, Table 1), and this was especially the case in children aged ≥5 years. In children aged <5 years, the mean IICD/IPD ratio was not significantly different between the two groups. The IICD/IPD ratio was not correlated with age (*r* = −0.032, p = 0.730, Fig 3A) in the epiblepharon group, but a negative correlation was observed in the control group (*r* = −0.248, p = 0.007, Fig 3B). For boys and girls, the mean IICD/IPD ratio was not significantly different between the two study groups. No difference was observed in the IICD/IPD ratio of boys and girls between either study group or in the entire cohort.

**Table 1:** IICD/IPD ratios by age and sex in the epiblepharon and control groups.

		IICD/IPD ratio, mean ± SD	95% CI	*P* value
Total	Epiblepharon (n = 119)	0.70 ± 0.04	0.69–0.70	<0.001
	Control (n = 119)	0.67 ± 0.04	0.67–0.68	
< 5 years old	Epiblepharon (n = 44)	0.70 ± 0.04	0.68–0.71	0.116
	Control (n = 44)	0.68 ± 0.03	0.67–0.69	
≥ 5 years old	Epiblepharon (n = 75)	0.70 ± 0.03	0.69–0.71	<0.001
	Control (n = 75)	0.67 ± 0.04	0.66–0.68	
Male	Epiblepharon (n = 58)	0.70 ± 0.04	0.69–0.71	<0.001
	Control (n = 58)	0.67 ± 0.04	0.66–0.68	
Female	Epiblepharon (n = 61)	0.70 ± 0.04	0.69–0.71	<0.001
	Control (n = 61)	0.67 ± 0.04	0.66–0.68	

IICD: inner intercanthal distance; IPD: interpupillary distance; CI: confidence interval.

The IICD/OICD ratio showed a similar pattern to that of the IICD/IPD ratio. The mean IICD/OICD ratio was higher in the epiblepharon group than in the control group (0.48±0.02 vs. 0.46±0.02, p<0.001, Table 2). The IICD/OICD ratio was higher in the epiblepharon group than in the control group only in those aged ≥5 years.

**Table 2:** IICD/OICD ratios by age and sex in the epiblepharon and control groups.

		IICD/OICD, mean ± SD	95% CI	*P* value
Total	Epiblepharon (n = 119)	0.48 ± 0.02	0.47–0.48	<0.001
	Control (n = 119)	0.46 ± 0.02	0.46–0.46	
< 5 years old	Epiblepharon (n = 44)	0.47 ± 0.03	0.46–0.48	0.633
	Control (n = 44)	0.47 ± 0.02	0.46–0.47	
≥ 5 years old	Epiblepharon (n = 75)	0.48 ± 0.02	0.47–0.48	<0.001
	Control (n = 75)	0.46 ± 0.02	0.45–0.46	
Male	Epiblepharon (n = 58)	0.47 ± 0.02	0.47–0.48	0.005
	Control (n = 58)	0.46 ± 0.02	0.45–0.47	
Female	Epiblepharon (n = 61)	0.48 ± 0.03	0.47–0.48	0.001
	Control (n = 61)	0.46 ± 0.02	0.45–0.46	

IICD: inner intercanthal distance; OICD: outer intercanthal distance; CI: confidence interval.

Epicanthal fold configurations were significantly different in the two study groups (Table 3). The type 2 configuration in which the upper skin fold forms a confluent fold with the lower eyelid at the interpalpebral region was more prevalent in the epiblepharon group than in the control group (p = 0.001), and this difference was significant for boys and girls. In analysis of age, the intergroup difference in epicanthal configurations was only significant for those aged ≥5 years (p = 0.001). In those aged <5 years, epicanthal fold configurations were not significantly different in the two study groups (p = 0.175).

**Table 3:** Types of epicanthal fold by age and sex in the epiblepharon and control groups.

		Type 1	Type 2	No epicanthus	*P* value*
Total	Epiblepharon (n = 119)	22	95	2	0.001
	Control (n = 119)	46	71	2	
< 5 years old	Epiblepharon (n = 44)	12	31	1	0.175
	Control (n = 44	18	25	1	
≥ 5 years old	Epiblepharon (n = 75)	10	64	1	0.001
	Control (n = 75)	28	46	1	
Male	Epiblepharon (n = 58)	8	49	1	0.009
	Control (n = 58)	20	37	1	
Female	Epiblepharon (n = 61)	14	46	1	0.032
	Control (n = 61)	25	35	1	

*Chi-square test.

The presence of an upper lid crease in the upper eyelid was not associated with epiblepharon. Thirty-six of the 119 patients with epiblepharon and 29 of 119 control children had such a crease (p = 0.38).

## Discussion

Congenital epiblepharon and epicanthus are common eyelid conditions among Asian children. The incidence and nature of epiblepharon has been documented in various Asian populations [7,8,14,15,16]. Epiblepharon is a common condition among East Asian children, which include, but are not limited to Chinese, Japanese, and Koreans [15]. In a study of 4449 Japanese children aged 3 months to 18 years, Noda et al. reported that the prevalence of epiblepharon was 10% [14]. This is similar to the prevalence of epiblepharon in a Singaporean study (9.5%) [16]. Epiblepharon is much less prevalent in Non-East Asian populations, such as Indians and Malays [15]. The epicanthal fold was extremely prevalent in our study population, regardless of the presence of epiblepharon. During the early stages of human embryogenesis, the eyes develop laterally on their optic stalks. From the fifth to ninth weeks, medial and anterior migration of the eyeballs rapidly occurs [17,18]. Lee et al. suggested that as the eyes move medially after formation of the eyelids, the initially concentric orbicularis oculi muscle converges at the medial canthal area to form an epicanthal fold [19]. This usually occurs at 3–6 months of gestation in all races and regresses with development [11]. Epicanthal folds are sometimes observed in infants of Western European descent with a low nasal bridge, but are rare after adolescence [20]. However, in most Asians, epicanthal folds remain a distinctive feature throughout the growing process, even until adulthood. These folds are due to an underdeveloped nasal root and an excess of horizontal medial canthal skin relative to a vertical skin shortage [19]. Obesity might be related to the pathogenesis or manifestation of epiblepharon. A high body mass index was observed in Japanese children with epiblepharon who were 6 to 11 years old [21]. Additionally, obese Korean girls aged 12 to 15 years demonstrated symptomatic epiblepharon at a significant level [22]. In the present study, the lack of a reduction in epicanthal severity with growth appeared to contribute to the pathogenesis of epiblepharon in children aged 5 years and older.

An elevated IICD/IPD or IICD/OICD ratio implies that the point of attachment of an epicanthal fold to the lower eyelids is located more laterally with respect to the medial canthal angle. Anatomically, dense connective tissue fibers originating from the medial canthal ligament run obliquely to the epicanthal fold [23]. Therefore, more lateral and anterior attachment of the epicanthal fold to the lower eyelids relative to the medial canthal angle can result in an upward and inward force to the lower eyelids. This augments rotation of cilia, which is observed in epiblepharon.

The configuration of insertion of epicanthus to the lower eyelids may also be related to formation of epiblepharon. Duke–Elder’s classification on epicanthus was not adopted in our study because it represents the general shape of the epicanthus and does not focus on the effect of the insertion pattern of epicanthus [24]. In the present study, type 2 epicanthus with a confluent fold appeared to drag more skin and orbicularis muscle upward from the lower eyelids to cause more severe lash inversion than that observed in type 1.

Epiblepharon tends to spontaneously disappear with age in Asian children [14]. In our control group, the severity of epicanthus was negatively correlated with age. This finding indicates that epicanthus decreases in severity with age. However, in the epiblepharon group, severe epicanthus persisted. This suggests that a lack of reduction in the epicanthus during growth in Asian children may be related to severe epiblepharon, requiring surgery for epiblepharon.

Medial propensity of epiblepharon has been described, but the reason for propensity has not been assessed. Kim et al. reported the results of surgical correction using an epicanthal weakening procedure in 101 patients with epiblepharon and an epicanthal fold [19]. They showed that preoperative medial propensity of keratopathy was in 87% of eyes. Our study suggests that the medial propensity of lower eyelid epiblepharon is related to the severity of epicanthus.

Recurrence of epiblepharon after surgical repair is also troublesome, especially at the medial portion of the lower eyelids [8,25,26]. Our team previously reported a 7.7% recurrence rate after epiblepharon surgery using a rotating suture technique [8]. Chang et al. reported a recurrence rate of 15.9% and an undercorrection rate of 9.1% after a modified Hotz procedure [26]. Various techniques have been used to treat epiblepharon [8,9,19,25,26]. However, regardless of the technique that is adopted, the medial most portion of the lower eyelids needs to be addressed to ensure successful surgery and prevent recurrence [19]. Combined epicanthoplasty is suitable for this purpose, but the resultant unsightly scar might be problematic for growing children. For medial correction of epiblepharon, great care should be taken to excise the proper amount of excessive tissue that is dragged by the epicanthal fold, avoiding canalicular damage.

One of the possible causative anatomical features of epiblepharon is the absence of adhesion between the lower eyelid retractors and the anterior lamella, thus allowing the skin and muscle to roll upwards. Therefore, we studied the configuration of the upper eyelid to determine the correlation between the presence of lower and upper eyelid epiblepharon designated by no crease. In the present study, the presence of an upper lid crease was not associated with epiblepharon of the lower eyelids.

The present study was performed using a retrospective design at a tertiary care center. Therefore, because of referral bias, the results are only applicable to severe cases that warrant epiblepharon surgery because mild cases were not included.

## Conclusion

This matched case–control study indicates a relationship between epicanthus and epiblepharon in East Asian children. Children that require epiblepharon surgery show a larger epicanthal skin fold that persists with growth compared with that observed in control children. Furthermore, the specific configuration of epicanthus forming a confluent fold with the lower eyelids has a higher prevalence in patients with epiblepharon than in controls. Finally, this study suggests that epicanthus can explain the medial propensity of epiblepharon and precipitate more severe epiblepharon, requiring corrective surgery.

## Supporting information

S1 Data RawData. (XLSX)
